# The Fasa Registry on Systolic Heart Failure (FaRSH): Feasibility Study and the First 5-year Reports

**DOI:** 10.31661/gmj.v10i0.2111

**Published:** 2021-09-02

**Authors:** Mohammad Hosein Yazdanpanah, Ehsan Bahramali, Maryam Kazemi, Negar Firouzabadi, Peyman Arasteh, Reza Homayounfar, Alireza Sepehri Shamloo, Mojtaba Farjam

**Affiliations:** ^1^Student Research Committee, Fasa University of Medical Sciences, Fasa, Iran; ^2^Non-communicable Diseases Research Center, Fasa University of Medical Sciences, Fasa, Iran; ^3^Digestive Disease Research Center, Digestive Disease Research Institute, Shariati Hospital, Tehran University of Medical Sciences, Tehran, Iran; ^4^Department of Community and Preventive Medicine, Fasa University of Medical Sciences, Fasa, Iran; ^5^Health Policy Research Center, Institute of Health, Shiraz University of Medical Sciences, Shiraz, Iran; ^6^Department of Pharmacology and Toxicology, School of Pharmacy, Shiraz University of Medical Sciences, Shiraz, Iran; ^7^Department of MPH, Shiraz University of Medical Sciences, Shiraz, Iran; ^8^Department of Electrophysiology, Heart Center Leipzig at University of Leipzig, Germany

**Keywords:** Heart Failure, Systolic, Registry, Iran

## Abstract

**Background:**

The literature on patients with heart failure (HF) from the Middle East, besides a few existing registries, is scarce. We report, for the first time in our country, a hospital-based registry for systolic HF.

**Materials and Methods:**

This was a web-based registry on HF, conducted in Vali-Asr Hospital affiliated with Fasa University of Medical Sciences, Fasa, Iran. The goal of this registry was to define overall baseline clinical characteristics and natural history of hospitalized patients with systolic HF, to evaluate current management schema and implementation of practice guidelines, and to determine the correlation between genetic predisposition environmental effects, individuals’ characteristics of health, lifestyle, morbidity, and mortality in relation with the effects of medication.

**Results:**

To date, 2378 individuals with a mean of age 67.08±13.07 years have been registered. Among which, 1381 (58.07%) patients were diagnosed with denovo HF. Most of the patients (60.1%) were male, and 8.9% had admissions during the past 30 days. The most common causes of HF were ischemic heart disease (86.5%) and hypertension (5.3%). Most patients had NYHA class one (44.3%) and three (20.4%). Overall, near 30% had diabetes and more than 38% had hyperlipidemia. Most individuals have been not a history of cigarette smoking (68.7%) or water-pipe smoking (96.9%). Also, 22.2% were current opium users, and 3.2% were previous opium users.

**Conclusion:**

The findings of this registry could make a realistic view of HF as a chronic disease with a burden. Therefore, policymakers can design programs and guidelines to prevent disease and better survival and quality of life.

## Introduction

Among the most common and disabling diseases of the 21 century is heart failure (HF). Despite significant advances in the management of the disease, it still affects an estimated 26 million individuals worldwide [1]. Managing the disease involves a multidisciplinary strategy, as the disorder is associated with a substantial decline in quality of life and is associated with many medical and social challenges [1,2]. Most guidelines and treatment protocols designed for patients with HF are based on data obtained from North America and Europe and by evaluating large registries, including the Acute Decompensated HF National Registry (ADHERE) [3] and the Organized Program to Initiate Lifesaving Treatment in Hospitalized Patients with HF (OPTIMIZE-HF) study [4]. Epidemiological data, which may affect protocol and treatment guidelines from other regions of the world, especially Asia, remains scarce [5]. Moreover, HF rates are increasing and among the most important factors contributing to the increased rates of HF is the transformation seen in the age pyramid, especially in Iran [6], as the population has transformed from a very young population to an older population [6]. Furthermore, due to advances in health care, survival after a cardiac event has increased over the past years, which itself increases HF rates [7,8]. Reports of patients with HF from the Middle East, besides a few existing registries, are scarce. This poses a major barrier for global collaborations and inhibits correct comparison of HF status and discussion on common issues in the management of HF. Here we report, for the first time in our country, a hospital-based registry for the evaluation of systolic HF and its correlates within our region.

## Materials and Methods

### Study Settings

This was a detailed description of our registry on systolic HF termed the Fasa Registry on Systolic HF (FaRSH, https://ncdrc.fums.ac.ir/activities/disease-registries/farsh) .This is a hospital web-based registry on HF, conducted in Vali-Asr Hospital as the first affiliated hospital of Fasa University of Medical Sciences (FUMS), Fasa, a city in Fras province, southeast of Iran, under the supervision of and funded by the Ministry of Health. The study was carried out by the Cardiology Department and the Noncommunicable Diseases Research Center (NCDRC) of FUMS. Vali-Asr Hospital is a university teaching hospital, fully equipped with chest pain units as well as coronary care unit (CCU), intensive care unit (ICU), catheterization lab, and cardiac surgery rooms for emergency interventions. It is a third-level referral center for 220 000 citizens residing in Fasa and 34 nearby towns and villages. One other medical center proximate to Fasa city also has general emergency departments. However, almost all patients with significant cardiovascular signs and symptoms are referred to Vali-Asr hospital for emergency and routine cardiac attention in about 600 000 subjects, including nearby cities and towns.

### Registry Objectives

The goals of this registry were (1) to define the overall baseline clinical characteristics and natural history of hospitalized patients with systolic HF in our region, (2) to evaluate the current management schema of systolic HF in the region, and to explore whether practice guidelines are implemented, (3) to evaluate further outcomes of patients with HF, and (4) to establish an infrastructure for further research and also their treatment on systolic HF. Other objectives include (5) to assess the correlation between treatment strategies and in-hospital and long-term outcomes, (6) to evaluate and compare the cost-effectiveness of medical and device therapies in HF, (7) and to determine the correlation between genetic factors and morbidity and mortality in relation with the effects of medication. Due to regulation of the Iranian communication law data gathering and handling were done. The study protocol was approved by the Research Board of FUMS (code: 93151) and the Ethics Committee for Biomedical Research at FUMS (code: E-9310).

### Patients

Patients entering the registry were classified into two groups: those diagnosed with acute new-onset HF (denovo HF) and individuals diagnosed with acute decompensation of chronic HF presenting with symptoms warranting hospitalization. The study was designed to include a five-year follow-up for each participant and a recruitment period from March 2015 to March 2020. All individuals included in the registry were considered based on admission and discharge diagnosis of systolic HF, which was made according to the International Classification of Diseases, Tenth Revision (ICD-10) coding [9] by the attending cardiologists who visited patients daily. All diagnoses were performed according to the European Society of Cardiology guidelines to diagnose acute and chronic HF [2]. Subjects with ejection fractions of less than 50% were considered in the registry. Patient recruitment began on March 8th, 2015, and was estimated to continue until March 2020, and the end of follow-ups is estimated to be on March 2025. The aims of the study were explained for patients followed by obtaining an informed consent

### Study Protocol

**Figure 1:**
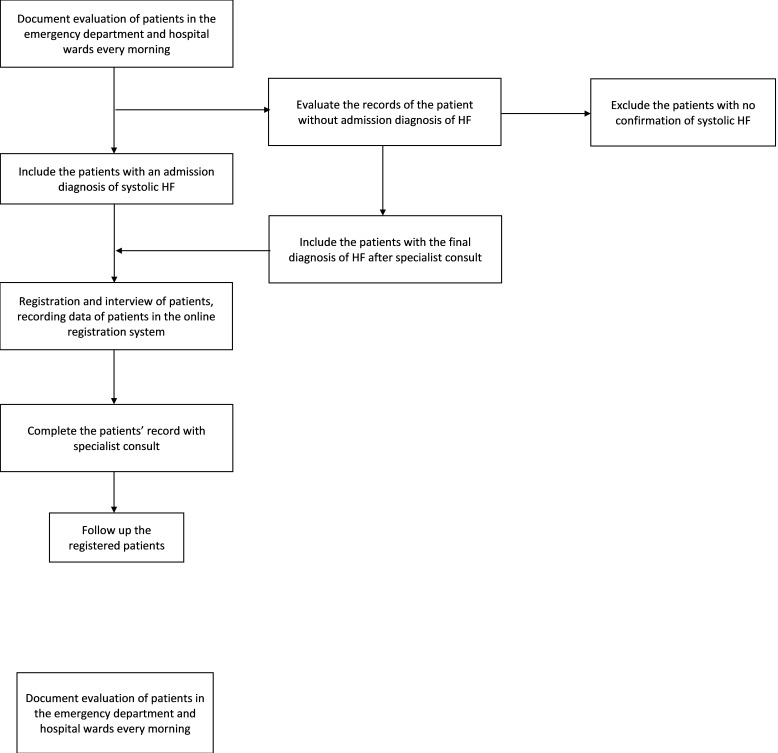
Flowchart of Fasa registry of systolic heart failure (FaRSH) design.

Parallel to the existing registry for acute myocardial infarction (MI)[10] and diabetes [11], for the registration of patients with systolic HF, two nurses were specifically trained for the evaluation of patients and registration of data. The nurses were trained by a single supervising cardiologist and five collaborating cardiologists for at least one month. The nurses were assigned to visit the hospital every morning at the start of the morning shift. They would first visit the emergency department and move on to the hospital wards, reviewing patients' documents and medical records. Admissions with a diagnosis of systolic HF would be included in the registry. Aside from evaluating patients' records, the nurses also conducted face-to-face interviews with each patient and/or their family to obtain data, according to the pre-designed data sheets and questionnaires. In cases where missing data existed, the primary attending physician who was responsible for that patient's treatment was available, and related data were obtained by referring to the treating physician.Registration was done electronically using an android tablet and through an online registration system. Throughout the university network framework, data were then processed on a cloud server that had stable firewalls. The flowchart of this registry design has been represented in Figure-1. Variables and Their Definitions The variables included in the study and their definition and classifications are reported in Table-1 and Table-2.

### Follow-up

After patients entered the registry, they were included in the electronic registration system. The online software, which included patients' information, would notify the registry nurses regarding each patient's follow-up appointments. After which, each patient or their relatives was contacted, and information regarding any cardiac or non-cardiac events was questioned. Specific questions were asked regarding cardiac interventions, other hospital admissions, events of bleeding, and stroke. In cases of any events, the patient or their relatives were interviewed regarding the event. In cases of any hospitalization or death, patients' medical records were reviewed and analyzed.

### Data Quality and Control

The electronic registration system notified the nurse who registers patients' data in cases of missing or unusual data entry. A working team of research assistants monitors and verify data entry weekly. The central database was further analyzed monthly by a steering committee, and completeness of data was assured. Data regarding the etiology of HF, including significant valvular heart disease and dilated cardiomyopathy (DCM), the existence of atrial fibrillation, reasons for cessation or decrease in the dose of drugs (such as angiotensin-converting enzyme [ACE] inhibitors, angiotensin receptor blockers [ARBs], beta-blockers, and mineralocorticoid receptor antagonists [MRA]) were entered in the registry database by validation of the attending cardiologist through the electronic system. Other data, including New York Heart Association (NYHA) class was measured and registered by the trained registry nurses who interviewed patients on their first day of admission.

### Statistical Analysis

Data analysis was done using the SPSS® software for windows®, version 17 (SPSS Inc., Chicago, IL, USA). Quantitative data were presented as mean±standard deviations (SD), and qualitative data were presented as frequency (percentage). Patients were classified into two groups of denovo HF and acute on chronic HF (ACHF). Means of normally distributed quantitative data were compared between the two groups using the independent T-test. Qualitative data were compared using the Chi-square test. A P-value of less than 0.05 was considered statistically significant.

## Results

Up to this date, 2378 individuals have been registered with the study. The mean age of patients was 67.08±13.07 years. A total of 375 (15.8%) patients have been diagnosed with denovo HF, 990 (41.6%) individuals were diagnosed with ACHF, 1005 (42.3%) patients for other causes admitted, and then HF was diagnosed during five years of the study. Patients mostly (60.1%) were males and from Fars and Arab backgrounds (69.1% and 18%, respectively). Almost 883 (37.1%) of the population lived in Fasa city. The mean body mass index (BMI) of the population was 24.73±6.33 kg/m2, and the mean waist circumference was 94.25±8.6 cm. The mean ejection fraction was 31.39±10.1, systolic blood pressure was 121.97±20.41, and diastolic blood pressure was 76.46±12.93. Also, the mean heart rate was 80.02±16.1.

Most patients had a previous hospital admission, among which 212 (8.9%) had a hospital admission within the past 30 days. The majority (53.3%) of patients had a history of HF from less than six months since their registration in the study. Regarding the etiology of HF, the most common causes were ischemic heart disease (86.5%), followed by hypertension (5.3%), and DCM (2.8%). Most patients had an NYHA class of one (44.3%) and three (20.4%) on registration. The majority (63.5%) of patients with HF had a positive history of MI. Atrial fibrillation was seen in 304 (12.8%) individuals. Past medical history showed that 277 (11.6%) of patients had heart valve disease, 156 (6.6%) had DCM (both in their previous echocardiograms), 185 (7.8%) had an episode of stroke, and 122 (5.1%) had chronic obstructive pulmonary disease. Coronary artery bypass graft (CABG) had been performed in 457 (19.2%), and percutaneous transluminal coronary angioplasty (PTCA) had been done in 615 (25.9%) of individuals with HF registered with the study (Table-3). Table-4 represents baseline characteristics of some HF-related risk factors for our population. Most individuals (68.7%) didn’t have a history of cigarette smoking or water pipe smoking (96.9%). Regarding opium inhalation, 529 (22.2%) were current opium users, and 76 (3.2%) of the population were previous opium users. Two of the registered individuals were alcohol users (except for six individuals whose status was unknown). Nearly half (48.7%) of the population who had hypertension were on oral medication, 74 (3.1%) were not using their medication, and 2 (0.1%) were on non-pharmacological treatments. Regarding diabetes, 481 (20.2%) were on oral medical therapy, 144 (6.1%) were on insulin, 40 (1.7%) were on oral medication and insulin, and 46 (1.9%) were not taking their medication. Overall, 775 (32.6%) of individuals who had hyperlipidemia were on oral medication, and 75 (3.2%) were not taking their medications. Table-5 displays specifics on medication use among patients with HF within the registry. The highest medication use was related to acetylsalicylic acid (ASA)-antiplatelets, with a 98.7% positive status of use among our population. Statins with 97.6% and anticoagulants with 96.6% were at the following ranks among medications. Among intravenous drugs, dopamine with 3.7% of the usage was in the first place. Also, digitalis with 14.2 % had the lowest frequency of usage among patients.

## Discussion

According to the global burden diseases study, cardiovascular disease (CVD) has been the major cause of death between 2010 and 2015 in Iran, accounting for more than 20% of all diseases and 46% of any and all mortality [12]. Previous reported data of the prevalence of CVD and its prediction in the Fasa region [13] signify the importance of heart (-related) diseases. Here we gave a detailed description of our registry on systolic HF in Iran termed the FaRSH. Aside from clarifying practice routines and patients’ appliance with treatment modalities, reasons for re-admissions, and medical treatment alterations, the FaRSH registry provides insight, for the first time, on systolic HF status in our country and to our knowledge, this is the first of its kind in Iran. We found that most individuals in our population were males, never smokers, never water pipe and opium users, and had a normal BMI. The majority of patients were referred due to symptoms of ACHF. In other words, most patients did not know that they had HF. Also, most individuals had no previous hospitalization, but in those with a previous history of hospitalization, most of them had an admission more than 30 days before, most patients had HF for less than six months. Near 52% had hypertension, near 30% had diabetes, and more than 38% had hyperlipidemia. Also, near 20% of individuals underwent CABG, and almost 26% underwent PTCA.

In Asia, more than ten registries exist on HF, which are mostly located in Japan [1,14,15], China [15,16,17], Taiwan [15,18,19], and South Korea [15,20]. Despite these efforts, data on HF from the Middle East is still largely missing, and only a few studies [21,22,23] have been conducted in this region on HF epidemiology in the context of a prospective HF registry. In the Euro HF registry, reported by Nieminen et al. [24], among 3580 individuals from 133 hospitals, they found a mean age of 69.9 years among their HF patients. Also, the Swedish HF registry reported that the mean of the included patients was 75 years (range 17–103) [25]. Moreover, in the ADHERE registry [26] that evaluated 105388 patients from 274 hospitals from across the United States, they found a mean age of 72.4 years among their patients with HF. This was significantly higher than that reported by the GULF registry (59 years old) [27]. We found the mean age of participants in our study was 67.08 years, this shows that Iranian patients are affected by HF in an age between neighboring countries (namely those in the GULF study) and that of Western countries.

Among the few studies that have been conducted in the Middle East, Sulaiman et al. [27] studied individuals with HF in 47 hospitals from the Middle East. In their study (the GULF registry), they found that 61% of their participants (vs. 52% in our study) had hypertension, 50% (vs. 30% in our study) had diabetes, 36% (vs. 38% in our study) had hyperlipidemia, and 22% (vs. 20.6% in our study) were smokers. Most of their patients were classified as NYHA class three and four (43% and 32%, respectively) [27], which in comparison to our study, most of our patients were classified into NYHA class one (44.3%), followed by class three (20.4%) was much different. Ischemia was considered the leading cause (53%) of HF, followed by hypertension (16%) [27]. In our study, the first leading cause of HF was ischemic heart disease with 86.5%, and the second one with 5.3% was hypertension. Another prospective registry from the Middle East is the heart function assessment registry trial in Saudi Arabia (HEARTS)[28], conducted in Saudi Arabia. Similar to the GULF registry[27], they found a lower mean age among participants with acute and chronic HF (60.6 and 56.9 years, respectively). Their population had higher BMI (29.3 and 29.2kg/m2 among individuals with acute and chronic HF, respectively), hypertension (70% and 75% in acute and chronic HF, respectively), hyperlipidemia (36.4% and 57.1% in acute and chronic HF, respectively), and diabetes (60.7% and 53% in acute and chronic HF, respectively). They found that 59% of their patients presented with NYHA classes three and four; furthermore, DCM (45%), coronary artery disease (40%), and hypertension (10.7%) were the most common causes of HF (aside from pregnancy-related cardiomyopathy) [28].

Overall, our HF patients seemed to have less severe conditions than the mentioned studies from the Middle East. This could be attributed to multiple factors. The first relates to the differences in baseline characteristics seen between the populations. Body fat mass, metabolic syndrome and its component, especially waist circumference and blood pressure, have been associated with electrocardiogram parameters and abnormalities [29,30] which may be factors associated to these differences in comparison to other populations. Second relates to the study sample selection between studies, as our study included individuals from a single center in Fasa city, and perhaps patients with more severe conditions are referred to centers in the main city of the province (Shiraz city). This study primarily was a report on protocol and preliminary findings and correct comparison with other studies from different regions may not be correct considering the main goal of the study and will be addressed in future upcoming studies. In addition, many of these studies included individuals who presented with either systolic or diastolic HF, while our registry only includes individuals with systolic HF. Thus, a precise comparison cannot be performed with most previous registries as some clinical characteristics of these conditions may differ. One of the most interesting findings was that almost 9% of our patients had a previous hospital admission during the last 30 days. This is alarming as it shows a flaw in the treatment process. Either patients were discharged too early and/or treatments were not followed by the individual as prescribed for them, which needs prompt attention and health care programming.

Other important findings were that ischemia was considered the primary cause of 86.5% of HF, CABG and PTCA were performed in 19.2% and 25.9% of individuals, respectively. This is alarming as it shows that many patients with HF who need cardiac interventions are being missed. If confirmed by future studies from other regions of the country, this presents a significant drawback in the management of HF and represents an important issue in our early intervention plans for HF. Underutilization of revascularization strategies in patients with systolic HF signifies an immediate need to increase cardiac catheterization and open-heart surgery facilities in the country or provide a more convenient route to access existing facilities for patients with HF. This registry records specifics on opium consumption, including dose, frequency, and type of opium. This provides an opportunity to study the associations between opium inhalation and HF, which is a subject of much controversy in literature [31,32,3]. Among other novelties in this registry, we further evaluated reasons for not prescribing HF medication or not prescribing the therapeutic dose of medications for each individual separately. This allows a precise evaluation of obedience to guidelines in the management of HF.

Drug consumption in our findings was another interesting point. ASA-antiplatelets drugs with 98.7% were at the first rank of medication use, followed by statins and anticoagulants, all with more than 90% usage. This broad use of medications in our region was surprisingly generally higher than other studies [34]. The reason for this wide use, the economic burden of this usage, and the compatibility of these drug administration with guidelines should be investigated further. The national health system absolutely would have benefited from the results of the further studies. Our health care system is divided into different health care sectors; they are each responsible for reporting to the medical university. This allows for a more detailed and reliable evaluation of the health status and offers a more effective means of collecting data in our research area. Therefore, the study follows up can be fortified with the help of health care workers in these sectors. 

Our registry includes more than 180 variables that are recorded from each patient. Data entry, as mentioned before, was performed by two dedicated and trained nurses under the supervision of a senior cardiologist; furthermore, data were re-evaluated weekly by a team of research assistants who are specifically trained for handling the data. As Vali-asr Hospital was assigned for data registry on systolic HF, attending cardiologists at the center assist in the precise diagnosis of the condition. Patients' personal data and course of the disease, including treatment modalities and outcomes are not allowed for disclosure for the general population, which certifies that treatment routines applied by the cardiologists at the medical care center would not be altered by the time the study is being conducted. Furthermore, since we had an observational method of data gathering, no specific treatment protocol depicted by the registry administrators was applied, thus not leading to any changes in routine treatments. Data acquisition included both medical history assessment by reviewing medical records and direct face-to-face interviews performed by the personnel, which increases the credibility of the data and compensates for the retrospective nature of data gathering seen in other registries. Our study has some limitations. Although clinical evaluation followed guideline-specific protocols, each individual's final diagnosis and treatment modalities were determined according to the treating physicians' clinical judgment. Also, our study was a single-center registry and may not be completely representative of the whole country. Unlike many previous registries on HF, we collected post-hospitalization clinical data, including revascularization, re-hospitalization, appliance to medical therapy, etc., that allows a precise evaluation of long-term outcomes. Our study only includes the territory covered by Fasa city. Furthermore, many individuals refer to Shiraz city (as the largest city in the Fars province) for medical care; therefore, the registry cannot claim to cover all hospitalized cases of HF within the region. This registry was the first of its kind in the country, and the study results will be used as a baseline study for further and larger upcoming registries.

## Conclusion

We are conducting the first hospital-based registry for systolic HF in Iran, which could help policymakers obtain a more realistic view of one of the most financially demanding chronic diseases in the country. We can define the costs of re-admissions, medications, invasive and non-invasive procedures, and devices used for patients with systolic HF and link them to their survival and quality of life.

## Acknowledgment

The authors would like to thank all personnel at the NCDRC and the cardiology department at FUMS for their collaborations with the study. The study was funded by FUMS (grant number: 93151). The funders did not play a role in any part of the study.

## Conflict of Interest

Authors have no conflict of interest to declare regarding any part of the manuscript.

**Table 1:** Variables Included in the Study

**Variables**	**Classification**
**Demographic**	Age, sex, height, weight, waist circumference, BMI, cigarette smoking, water pipe smoking, opium use, alcohol use, ethnicity, and place of residence
**HF-related data**	Date of admission, date of discharge, the reason for admission, date of last hospital admission, duration of heart failure, primary etiology of heart failure, NYHA score, history of previous MI, systolic and diastolic blood pressure
**Other diseases**	Atrial fibrillation, COPD, valve-related disease, dilated cardiomyopathy, stroke, cancer, dementia, previous and current hypertension, previous and current diabetes, previous and current hyperlipidemia
**Interventions**	CABG, PTCA, revascularization, the existence of defibrillator devices
Medications*	ACE inhibitors, ARB, beta-blockers, MRA, statins, diuretics, inotropic medications, anticoagulants, long-acting nitrates, antiplatelet medications, and other non-cardiac related drugs
**Family history of diseases**	MI, stroke, heart failure and the relationship between the individual with these conditions and the patient
**Lab data**	Serum glucose, total cholesterol, HDL, LDL, FBS, and TG

**HF:** Heart failure;** BMI:** Body mass index;** NYHA:** New York Heart Association;** MI: **Myocardial infarction;** COPD:** Chronic obstructive pulmonary disease;** CABG: **Coronary artery bypass grafting;** PTCA: **Percutaneous transluminal coronary angioplasty;** ACE:** Angiotensin-converting enzyme;** ARB:** Angiotensin receptor blockers;** MRA: **Mineralocorticoid receptor antagonists; **HDL:** High-density cholesterol; **LDL:** Lowdensity cholesterol;** FBS: **Fasting blood sugar;** TG: **Triglyceride. *All medications were recorded upon discharge; however, the use of diuretic medications during admission was also registered.

**Table 2:** Definition/Classifications of the Variables

**Variables**	**Definition/Classification**
**Reason of admission**	Worsening of symptoms (acute or chronic HF or ACHF), new diagnosis of HF (denovo HF)
**Ethnic groups**	Arab, Fars, Lor, Tork, and unknown
**Place of residence**	Living in Fasa city, living in a village, from outside of Fasa territory
**Previous admissions due to HF**	Admission less than 30 days ago, more than 30 days ago, without a previous admission
**Duration of HF**	Less than six months, more than six months
**Etiology of HF**	Hypertension, ischemic heart disease, DCM, alcoholic cardiomyopathy, valve disorders, right ventricular failure, unknown and other causes
**Hypertension**	A systolic blood pressure of ≥140 or a diastolic blood pressure of ≥90
**Hypertension treatment**	Not receiving any treatment, those on non-pharmacological treatment (like traditional medicine), those on oral medications
**Hyperlipidemia**	TG>200mg/dl or total cholesterol of >240mg/dl
**Hyperlipidemia treatment**	Not on any treatment, on diet control, on oral medication, or unknown treatment.
**Diabetes treatment**	Not receiving treatment, those on diet control, on oral medication, on insulin, or finally on treatment that was unknown
**Cigarette, water pipe, and opium user**	Current smokers, ex-smokers, and never smokers (An individual who smoked 100 cigarettes in his/her life was considered a smoker. For water pipe and opium, only history of regular use was considered when labeling individuals as users)
**Type of opium**	Opium, opium extract (or Shire), and other types
**Dose of opium**	Less than once a week, 1-4 times a week, 5-9 times a week, 10-14 times a week, and more than 14 times a week
**Previous episodes of MI**	Documented or non-documented

**HF:** Heart failure;** ACHF:** Acute on chronic heart failure;** DCM:** Dilated cardiomyopathy;** MI:** Myocardial infarction;** TG:** Triglyceride

**Table 3:** Baseline and HF-Related Characteristics in the Fasa Registry on Systolic HF

**Variables**	**Groups**	**ACHF**	**Denovo AHF (n=1381)**	**Total**	**P-value**	
		**(n=990)**		**(n=2371)**	
**Age, year**		70.4±11.66	64.73±13.4	67.08±13.07	<0.001
**Gender**	Male	525 (36.8)	900 (63.2)	1430 (60.1)	<0.001
	Female	465 (49.2)	481 (50.8)	948 (39.9)	
**Ethnicity**	Arab	227(53.2)	200 (46.8)	427 (18)	<0.001
	Fars	627 (38.3)	1009 (61.7)	1643 (69.1)	
	Lor	5(31.3)	11 (68.8)	16 (0.7)	
	Tork	130 (45.5)	156 (54.5)	286 (12)	
	Unknown	1 (50)	1 (50)	2 (0.1)	
**Location of residence**	Fasa	454 (51.6)	425 (48.4)	883 (37.1)	<0.001
	Village	426 (51.6)	400 (48.4)	828 (34.8)	
	Outside Fasa territory	110 (16.5)	556 (83.5)	667 (28)	
**BMI, kg/m2**		24.54±4.37	24.85±7.31	24.73±6.33	0.2
**Waist circumference, cm**		94.9±9.99	93.82±7.55	94.25±8.6	0.01
**Previous hospital admission due to HF**	No	31(2.6)	1176(97.4)	1207(50.8)	<0.001
	<30days	170 (80.2)	42 (19.8)	212 (8.9)	
	>30days	784 (82.8)	163(17.2)	947 (39.8)	
**Duration of HF**	<6months	79 (6.2)	1188(93.8)	1267 (53.3)	<0.001
	>6months	906 (82.4)	193 (17.6)	1099 (46.2)	
**Etiology of HF**	Ischemic heart disease	800 (38.9)	1257 (61.1)	2058 (86.5)	<0.001
	Hypertension	93 (73.8)	33 (26.2)	126 (5.3)	
	Dilated cardiomyopathy	52 (77.6)	15 (22.4)	67 (2.8)	
	Valve disorders	61 (70.1)	26 (29.9)	87 (3.7)	
**NYHA class**	1	98 (9.3)	954 (90.7)	1053 (44.3)	<0.001
	2	241 (57.7)	177 (42.3)	418 (17.6)	
	3	346 (71.2)	140 (28.8)	486 (20.4)	
	4	235 (76.1)	74 (23.9)	309 (13)	
**CABG**	Yes	287 (62.9)	169 (37.1)	457 (19.2)	<0.001
	No	696 (36.5)	1211 (63.5)	1908 (80.2)	
**PTCA**	Yes	325 (52.9)	289 (47.1)	615 (25.9)	<0.001
	No	656 (37.6)	1091 (62.4)	1748 (73.5)

**ACHF:** Acute on Chronic heart failure;** AHF:** Acute heart failure;** HTN:** Hypertension; **TX:** Treatment

**Table 4:** Baseline Characteristics on HF-Related Risk Factors

**Variables**	**Groups**	**ACHF (n=990)**	**Denovo AHF (n=1381)**	**Total**	**P-value**	
		**n (%)**				
**Smoking**	Current	155 (31.7)	334 (68.3)	489 (20.6)	<0.001	
	Ex-smoker	124 (51.4)	119 (48.6)	245 (10.3)		
	Never	703 (43.1)	928 (56.9)	1633 (68.7)		
**Water pipe**	Current	4 (11.8)	30 (88.2)	34(1.4)	<0.001	
	Ex-smoker	15 (51.7)	14 (48.3)	29 (1.2)		
	Never	965 (41.9)	1334 (58.1)	2304 (96.9)		
**Opium use**	Current	181 (34.3)	347 (65.7)	529 (22.2)	<0.001	
	Ex-smoker	27 (36)	48 (64)	76 (3.2)		
	Never	755 (43.5)	980 (65.5)	1736 (73)		
**Hypertension**	Yes, and not on TX	31 (41.9)	43 (58.1)	74 (3.1)	<0.001	
	Yes, and on non- pharmacological TX	1 (50)	1(50)	2 (0.1)		
	Yes, on oral medication	564 (48.7)	593 (46.7)	1158 (48.7)		
	No	388 (34.3)	744 (65.7)	1132(47.6)		
**Diabetes**	Yes, and not on TX	17 (37)	29(63)	46 (1.9)	<0.001	
	Yes, and on diet control	8 (42.1)	11 (57.9)	19 (0.8)		
	Yes, and on oral medication	232 (48.2)	249 (51.8)	481 (20.2)		
	Yes, and on insulin	87 (60.4)	57 (39.6)	144 (6.1)		
	No	621 (38)	1013 (62)	1634 (68.7)		
**Hyperlipidemia**	Yes, and not on TX	26 (34.7)	49 (65.3)	75 (3.2)	<0.001	
	Yes, and diet control	21 (51.2)	20 (48.8)	42 (1.8)		
	Yes, and on oral TX	425 (54.8)	350 (45.2)	775 (32.6)		
	No	511 (34.7)	962 (65.3)	1473 (61.9)

**ACHF: **Acute on Chronic heart failure; **AHF:** Acute heart failure; **HTN: **Hypertension; **TX:** Treatment

**Table 5:** Medication Use Amongst the First 5-years Population with Systolic HF.

**Variables**	**Groups**	**ACHF**	**Denovo AHF**	**Total**	**P-value**
		**(n=990)**	**(n=1381)**		
		**n (%)**			
**ACE-inhibitor**	Yes	651 (38.3)	1047 (61.7)	1699 (71.4)	<0.001
	No	335 (50.1)	334 (49.9)	670 (28.2)	
**ARB**	Yes	244 (50.9)	235 (49.1)	479 (20.1)	<0.001
	No	743 (39.3)	1146 (60.7)	189 (79.5)	
**Beta-blocker**	Yes	826 (39.5)	1267 (60.5)	2095 (88.1)	<0.001
	No	160 (58.4)	114 (41.6)	274 (11.5)	
**MRA**	Yes	652 (53.1)	577 (46.9)	1229 (51.7)	<0.001
	No	334 (29.3)	804(70.7)	1140 (47.9)	
**Spironolactone or eplerenone**	Yes	624 (52.4)	567 (47.6)	1191 (50.1)	<0.001
	No	365 (31)	814 (69)	1187 (49.9)	
**Diuretic**	Yes	746 (62.9)	440(37.1)	1187 (49.9)	<0.001
	No	239 (20.3)	940 (79.7)	1181 (49.7)	
**Digitalis**	Yes	288 (85.5)	49 (14.5)	337 (14.2)	<0.001
	No	696 (34.4)	1332 (65.7)	2030 (85.4)	
**Statin**	Yes	950 (41)	1368(59)	2320 (97.6)	<0.001
	No	36 (73.5)	13 (26.5)	49 (2.1)	
**Intravenous-inotropic**	Dobutamine	34 (73.9)	12 (26.1)	46 (1.9)	<0.001
	Dopamine	40 (44.9)	49 (55.1)	89 (3.7)	
	Levosimendan	2 (50)	2(50)	4 (0.2)	
	No	898 (40.8)	1303 (59.2)	2203 (92.6)	
**Long-acting nitrate**	Yes	693 (47.8)	758 (522)	1453 (61.1)	<0.001
	No	294 (32.1)	622 (67.9)	916 (38.5)	
**Anticoagulant**	Yes	944 (41.1)	1352 (58.9)	2298 (96.6)	<0.001
	No	38 (56.7)	29 (43.2)	67 (2.8)	
**ASA-antiplatelet**	Yes	976 (41.6)	1369 (58.4)	2347 (98.7)	<0.001
	No	10 (45.5)	12(54.5)	22 (0.9)

**ACHF:** Acute on chronic heart failure; **AHF: **Acute heart failure;** ACE:** Angiotensin-converting enzyme;** ARB:** Angiotensin II receptor blockers;** MRA:** Mineralocorticoid receptor antagonists;** ASA: **Acetylsalicylic acid.
